# Social normative and social network factors associated with adolescent pregnancy: a cross-sectional study of 176 villages in rural Honduras

**DOI:** 10.7189/jogh.10.010706

**Published:** 2020-06

**Authors:** Holly B Shakya, Gary L Darmstadt, Kathryn M Barker, John Weeks, Nicholas A Christakis

**Affiliations:** 1 Department of Medicine, University of California San Diego, La Jolla, California, USA; 2 Department of Pediatrics, Stanford University School of Medicine, Stanford, California, USA; 3 San Diego State University, San Diego, California, USA; 4 Yale University, New Haven, Connecticut, USA

## Abstract

**Background:**

Adolescent pregnancy and childbirth are common throughout Central America. While gendered beliefs promoting motherhood are a known risk factor, their association with adolescent childbirth within the social networks of Central American communities is unknown.

**Methods:**

This was a cross-sectional study looking at adolescent childbirth amongst women ages 15-20 years (N = 2990) in rural Honduras, using reproductive health data on all individuals ≥15 years of age (N = 24 937 of 31 300 population) including social network contacts, all of whom were interviewed as part of the study. The outcome, adolescent childbirth, was defined as having had a child < age 20 years. Predictors included whether a woman’s social contact had an adolescent childbirth and the social contact’s reported perception of community support for adolescent childbirth.

**Results:**

While girls who identified a father in the village as a social contact had a lower likelihood of adolescent childbirth regardless of whether or not they reported being in a partnership, this finding did not hold for girls who identified mothers. There was an association between a social contact’s report of norms supporting adolescent childbirth and a girl’s risk of adolescent childbirth; however, village-level aggregate norms attenuated that relationship. Independent significant associations were found between a girl’s risk of adolescent childbirth and both a social contact’s adolescent childbirth and the village proportion of women who had had an adolescent childbirth. The association between social contacts’ adolescent childbirth and a girl’s risk of adolescent childbirth across relationships was more robust for stronger relationships and when the social contact was closer in age to the girl.

**Conclusions:**

If, as this evidence suggests, a strong driver of adolescent childbirth is the frequency of the occurrence of adolescent childbirth both within the greater community and within a girl’s proximal social network, the challenge for intervention strategies is to encourage norms that prevent adolescent childbirth without stigmatising those who have had an adolescent childbirth. Programmatic efforts to counter prevailing norms that limit a woman’s role to motherhood, and that support and encourage strong norms for girls’ education may play an important role in addressing this situation.

Rates of adolescent pregnancy in Latin America and the Caribbean are higher than for other regions with similar levels of development [1,2]. Honduras has the highest adolescent birthrate in Central America, at 137 births for every 1000 women ages 15 to 19 years old [3]. According to data from the most recent Honduras Demographic and Health Survey (DHS) (2011-2012), nearly one quarter of Honduran girls in this age group were either pregnant or a mother [4]. Consistent with determinants of adolescent pregnancy in other places, the risk increases for girls in rural areas; with lower levels of education; who grow up in unstable family environments where there may be no father [5]; where there is family violence or migration [6,7]; and who are living in poverty [6,8,9]. Girls who experience an adolescent birth become part of the “poverty reproduction cycle”[10], in which impoverished girls are at higher risk for an adolescent pregnancy, which in turn limits the possibility that the girl and her offspring will escape the poverty that was a key determinant of the pregnancy. This is specifically true for girls, as the burden of early childbearing disproportionately falls on mothers compared to fathers. Another key consequence for girls who become mothers is the experience of gender bias, and the inability to escape limited gender-restricted roles [10], as motherhood itself feeds into gender norms of sacrifice of self to family, high levels of domestic obligation or unpaid work, and economic dependence. Similar to the perpetuation of poverty, adolescent childbirth becomes both a consequence and driver of gender inequality. Overall, adolescent fertility is associated with poor physical and mental health outcomes, as well as enduring social and economic disadvantages for individuals, their families, and nations [11-13].

## Adolescent childbearing: Theoretical frameworks

Interconnected groups of individuals can shape the health-related behaviour of individuals by transmitting and enforcing group-specific social norms [14]. Social norms are unwritten rules and informal understandings that prescribe (support) and proscribe (prohibit) behavioural regularities in groups and societies [15,16]. Innumerable health behaviour theories emphasise the importance of norms in shaping behaviours [17-19]. Work by Cialdini and colleagues has identified several key constructs in norms theory that are relevant for health behavior [20]. Descriptive norms are defined as the regular and observable behaviours of persons in the social milieu and can signal what actions are acceptable vs deviant or off-limits [21,22]. Injunctive norms are an individual’s perceptions of which behaviours meet the approval of others [23]. Compliance with injunctive norms can facilitate social inclusion, while noncompliance can trigger social sanctions, such as ostracization, loss of respect, and derision.

Social norms operate within reference groups, or the groups of people to whom an individual turns for expectations regarding what is common and what is acceptable [14,24,25]. Reference groups may comprise proximal relationships such as close friendships, social communities that are an extension of proximal relationships, or a wider community such as a village [14]. Identifying reference groups can be crucial for intervention efforts because if the intervention message counters prevailing norms, those norms may hinder the success of that intervention [26]. Many health behaviour interventions have failed because programs were designed to intervene on individuals with no regard for the greater social context [27]. Identifying normative barriers and developing strategies to work for normative change within relevant groups is a key strategy for implementing successful health behaviour interventions.

There is evidence that social factors play a role in adolescent childbearing. Both injunctive and descriptive pregnancy norms of girls’ close friends and family members have been shown to influence their likelihood of adolescent childbirth [28,29]. However, the social factors impacting adolescent childbearing are complex, as they can differ greatly depending on the context. In regions with mostly low social control of adolescent sexual behavior, like Latin America and some parts of Sub-Saharan Africa, adolescent childbearing is often the result of unregulated sexual activity outside of marriage [30]. This contrasts with cultural contexts such as India or other regions of Sub-Saharan Africa where adolescent childbearing is the result of marriages that have been arranged by parents to protect family honor [31]. In the former, descriptive norms could be considered the primary driving force behind adolescent pregnancy outcomes, with adolescent girls making decisions based on what they see is acceptable in their communities, whereas in the latter, injunctive norms may matter more, with parents making marriage decisions for adolescent girls based on their own perceptions of societal expectations [31,32].

While adolescent childbearing in Central America is often associated with low social control of adolescent sexuality, there is evidence that injunctive norms around motherhood may play a role as well. Prevailing norms in the Central American region link women’s status to their fertility [33]. Previous work has noted that cultural norms surrounding motherhood likely support and encourage early unions and childbearing [1], and girls who live in communities with a strong cultural emphasis on motherhood are at higher risk of adolescent childbearing [34]. On the other hand, the risk of adolescent childbearing has been shown to decrease within communities and families that hold strong norms against early pregnancy and that are socially cohesive [7,35,36].

Despite the importance of seeking to understand the impact of an adolescent’s social network in the context of studies of adolescent fertility, studies typically focus on individual-level risk behaviours [37]. Research examining socio-contextual factors has been largely qualitative, as most population-level surveys such as DHS lack contextual-level and social normative measures [38]. Given these gaps, the degree to which social network and social normative factors are associated with adolescent birth remains unclear.

Using a novel data set from rural Honduras, this study analyses the social network correlates of adolescent childbirth. Here we use data from a high adolescent fertility region of Honduras on social network relationships, individual attitudes, perceived community norms, and descriptive norms specific to adolescent childbirth to generate evidence about the norms that may be most strongly associated with adolescent childbirth, and the salient reference groups for those norms.

## METHODS

### Study population

Our study uses full population census data from the western municipalities of the largely rural Copán department of Honduras to analyse the social network determinants of adolescent childbirth at the individual level. Data were collected as part of a randomised controlled trial of social network targeting of a maternal and neonatal health intervention in a study area comprised of 176 villages located in the municipalities of Copán Ruinas, Santa Rita, Cabañas, and San Jerónimo [33,39]. The area was chosen because the villages were geographically diverse, the population experiences high vulnerability to maternal and neonatal health complications, and the location was suitable for data collection. A full description of the study design and data collection methods is published elsewhere [39]. This part of Honduras also has a traditionally high rate of adolescent fertility [4], making it an ideal study setting for understanding the social dynamics around adolescent childbirth.

We mapped the study area to gain a more precise profile of the study population and field conditions, including terrain, rainfall, and distances to health facilities. This is an area of over 200 square miles of rugged mountainous terrain with an estimated total population of 32 800 people older than 12 years of age. We conducted a complete census in 2016 which covered 92% of the eligible population. Of these, 25 032 completed a baseline survey that included sociocentric network and behavioural health measures. For the purposes of these analyses, we excluded children under age 15 (N = 2577) as they did not complete the full reproductive history, and the proportion who had already experienced a childbirth was miniscule. Individuals who were cognitively impaired and unable to provide consent were also excluded (N = 30). With the above exclusions, our final number of study participants was 22 449.

### Network data collection

We used the publicly available software “Trellis” (http://humannaturelab.net/resources/software/trellis/) to undertake the main survey, which included a battery of “name generator” questions to capture social relationships. We took photographs of all people from whom data were collected in this study, so that individuals could identify their social contacts by picture. Individuals were notified at the beginning of the study regarding the methods to be used, including photographs to be used for network identification. All participants had the option of refusing to have their photo taken. Network identification was anonymized within the data files and encrypted. The boundaries of each network were the village, so that individuals could nominate any individual from within their own village as a social contact.

### Measures

Our unit of analysis was restricted to individual adolescent girls between the ages of 15 and 20 at the time of the survey, for whom an adolescent birth would have been within the last three years (N = 2990). The outcome, adolescent childbirth, was defined as having had a child under the age of 20, consistent with the definition used in DHS and other similar demographic surveys. Social network measures for adolescent girls were calculated based on the full village network. All individuals within each village were interviewed about their health and reproductive history as well as their social networks. Adolescent girls were matched with the people they identified (termed here, alters), so that we could assess the correlations between alters’ characteristics and adolescent childbirth outcomes. Village-level measures included aggregates of the entire study population by village.

#### Outcome variable: adolescent childbirth

Female respondents were asked whether or not they had ever given birth to a living child, and if so they were then asked to provide the birthdates of their last four children. For adolescents with four or fewer children, their age at first birth was calculated as the difference between their date of birth and the date of birth of their first child. None of the adolescents in our primary sample population of females ages 15-20 had more than 4 children.

#### Individual demographics

Individual demographic variables included age, marital status, religion, income sufficiency, education, food security, proportion of life lived in the village, and indigenous status. For exact coding of these measures, (see Appendix S1 in the **Online Supplementary Document** for specific questions).

#### Attitudes and social norms at the individual level

All respondents were asked their personal attitude regarding the appropriate age of first birth for women, “At what age is it OK for a girl to have her first baby?” We categorised personal attitudes as “Below the age of 20”, “20 and above”, and “Don’t know”. Analyses were run using “below the age of 20” as the comparison group.

We also asked each respondent regarding injunctive norms around adolescent birth: “If a girl younger than 18 has a baby, will people in the community think this is good, bad, or neither?” We modeled normative beliefs in support of adolescent birth as a binary variable, “Good” or “Bad/Neither”, as the statistical model showed no difference in the association between Bad or Neither with adolescent birth, but a strong difference between Good and Bad/Neither. In this case, coding the variable as continuous would have produced an artificial result suggesting a linear relationship.

### Reference groups

#### Village-level normative factors

To investigate the potential impact of a reference group that exists at the community level we used village level aggregate normative measures. We calculated the proportion of people in each village, including every individual that was interviewed in each village, that reported perceptions of norms regarding birth under the age of 18 as “Good”. This measure, which we term collective injunctive norms, allows us to understand norms as reflective of the greater social environment, beyond just the individual [40-42]. As a proxy for descriptive norms, which would ideally be measured by asking each respondent what they think is normally practiced, we calculated the proportion of all women in each village who had a birth under 20 (descriptive norms). Because adolescent childbirth is not something that can be easily hidden within a community, an aggregation of adolescent childbirth at the village level can give us a reasonably accurate idea of what individuals in the community would perceive to be the normative practice [43].

#### Social network data collection

Sociocentric studies attempt to ascertain all of the social relationships within a defined population (Marin & Wellman, 2011). Here, we defined the full networks at the village level. All participating respondents in the village took part in the network survey, and their nominations could include anyone within the village. Respondents were asked 14 separate *name generator* questions regarding their social connections to individuals within the community, including familial relationships, close personal relationships, economic support, and health advice (see Appendix S1 in the **Online Supplementary Document** for specific questions). From these questions we were able to ascertain the full networks of each village, allowing us to calculate individual network characteristics for each respondent.

#### Alter-level factors

In order to understand the potential association of a reference group at the more proximal interpersonal level, we used measures from individual girls’ direct social network ties, as described above. Because all questions asked of the adolescent girls in our study were asked of everyone in the study population, we were able to match each adolescent with the social normative factors, including beliefs, attitudes, and adolescent birth outcome of each individual she nominated in the social network survey (alters), as well as with those who nominated her. An individual could name any one alter up to 14 times through the name generators. We therefore have data for each of the adolescents’ social connections, and we know through which questions they were named. For each adolescent girl-alter pair, we calculated a measure of *tie strength*, or a simple count of the number of name generators in which the adolescent had nominated that particular alter, which can serve as a proxy for how close they are to each other. We also created a measure of *direction*. Did the adolescent girl nominate the alter (*out-ties*), did the alter nominate the girl *(in-ties)*, or was the nomination reciprocal *(both-ties).*

### Statistical analyses

We ran analyses at the individual level and at the dyadic level of social network connections. Individual analyses used logistic regression with the outcome being an adolescent birth, and were run using multilevel modeling, clustered at the village level. For our first set of models, we tested whether family composition, namely nominating a mother or a father in the village, was associated with having had an adolescent childbirth. All models controlled for sociodemographic characteristics, including time spent in the village. We then ran a series of models using dyadic observations, using logistic regression with adolescent birth as the outcome. Each adolescent girl-alter pair comprised one observation, with controls for girl’s age, education, religion, indigenous status, income insecurity, years lived in village, and village size. For these models, we were able to ascertain whether alter-level characteristics were associated with the likelihood that a girl had an adolescent childbirth. Our first set of models was run using the adolescent girl’s full network, including those she had nominated and those who had nominated her. Our second set of models was focused on those the girl herself had nominated and were stratified by name generator in order to pinpoint potential reference groups for normative beliefs and behaviours associated with adolescent childbirth. All dyadic level models were run using general estimating equation functions to adjust for multiple observations of the same girl.

## RESULTS

### Descriptive statistics

**Table 1** and **Table 2** show the descriptive statistics of the study population of adolescent girls (**Table 1**) and the alters nominated by those girls (**Table 2**). Of the girls in this study, 31% had an adolescent childbirth. Compared to girls who had not had an adolescent childbirth, those who had an adolescent childbirth were older (18.4 vs 16.9 years), less educated (4.2 vs 5.2 years of schooling) and had lived in the village for a shorter period of time (11.5 vs 14.1 years). Alters of girls who had had an adolescent pregnancy compared to alters of girls who had not were more likely to have had an adolescent childbirth themselves (41% vs 29%), equally likely to be male (41% vs 42%), and less likely to be from the same household (21% vs 34%).

**Table 1:** Descriptive characteristics of adolescent girls under the age of 21 in 176 villages in rural Honduras

	No adolescent pregnancy ego characteristics N = 2065	Adolescent pregnancy ego characteristics N = 914
	**% or mean (SD)**	**% or mean (SD)**
Named a spouse	17%	79%
Named a father	69%	40%
Named a mother	84%	54%
Education	5.25 (1.9)	4.2 (2.2)
Income	2.83 (0.80)	2.68 (0.76)
Indigenous	10%	10%
Food	0.48	0.65
Catholic	54%	47%
Protestant	35%	37%
No religion	11%	17%
Year in village	14.1 (5.6)	11.2 (7.5)
Age	16.9 (1.6)	18.4 (1.4)

SD – standard deviation

**Table 2:** Descriptive characteristics of alters nominated by adolescent girls under the age of 21 in 176 villages in rural Honduras

	No adolescent pregnancy alter characteristics N = 14336	Adolescent pregnancy alter characteristics N = 6352
	**% or mean (SD)**	**% or mean (SD)**
Age	34.4 (15.7)	36.3 (15.1)
Sex Male	42%	41%
Has had an adolescent birth	29%	41%
Believes community supports adolescent birth	13%	16%
Believes in younger age for first pregnancy	15%	20%
Tie strength	1.29 (1.3)	1.29 (1.3)
Personal private	21%	21%
Free time	25%	25%
Closest friend	26%	26%
Same household as ego	34%	21%

SD – standard deviation

### Individual analyses: familial nominations

Our first set of analyses at the individual level tested whether familial relationships were associated with an adolescent childbirth outcome. **Table 3** shows the results of two sets of models. In Model 1, we found that having nominated a mother in the village and having nominated a father in the village were both significantly associated with a decreased likelihood of having had an adolescent childbirth. As it is possible that girls who have had an adolescent childbirth did so after having moved away from their parents, our models were adjusted for the time spent in the village. To further test the potential influence of girls leaving home with a partner, Model 2 included a measure of whether or not the adolescent girl identified having a partner in the village. The relationship of nominating a mother and a girl’s adolescent childbirth outcome disappeared with the inclusion of a partner; however, the relationship of nominating a father and a girl’s adolescent childbirth remained robustly significant. Setting all parameters to their mean, the predicted probability (PP) of having had an adolescent childbirth for adolescent girls with a spouse and a father was 46% (95% confidence interval (CI)  = 40%-52%) compared to 58% (95% CI = 51%-65%) for a girl with a spouse and no father, while the PP of having had an adolescent childbirth with a spouse and no mother was 43% (95% CI = 34%-52%) compared to 46% (95% CI = 40%-52%) for having a spouse and a mother.

**Table 3:** Individual level and family characteristics associated with having had an adolescent pregnancy among girls less than 21 y of age

	Model 1			Model 2		
	**Beta**	**SE**	***P*-value**	**Beta**	**SE**	***P*-value**
Named a spouse				2.6	0.13	0
Named a father	-0.58	0.12	0	-0.48	0.14	0
Named a mother	-0.58	0.15	0	0.12	0.18	0.51
Education	-0.21	0.02	0	-0.14	0.03	0
Income	-0.17	0.07	0.01	-0.23	0.08	0
Indigenous	0.02	0.18	0.92	0.02	0.2	0.93
Food	0.13	0.05	0.01	0.17	0.06	0
Religion	0.2	0.15	0.17	0.16	0.16	0.32
Religion	0.21	0.11	0.06	0.23	0.12	0.06
Year in village	-0.03	0.01	0	0.01	0.01	0.3
Age	0.61	0.03	0	0.52	0.04	0
AIC	2769			2280		
ICC	0.048			0.038		

SE – standard error, AIC - Akaike information criterion, ICC – inter-class correlation coefficient

### Dyadic analyses

#### Adolescent girls’ full network

**Table 4**, **Table 5** and **Table 6** show the results of dyadic analyses which considered the association of an adolescent girl’s childbirth with characteristics of her alters, including alter’s childbirth and reported norms. Analyses in **Table 4** were created using the entire network of relationships based on 13 name generators (excluding children over age 12 living outside the house), including the people who nominated her and the people whom she nominated. In Model 1 we see that a girl’s likelihood of an adolescent childbirth outcome was significantly higher if the alter had an adolescent birth (PP 26% (95% CI = 25%-27%) compared to 17% (95% CI = 16%-17.5%) if the alter did not have an adolescent birth) or if the alter believed that the community approves of adolescent childbirth (PP 23%, 95% CI = 21%-25%) compared to 19% (95% CI = 18%-20%) if the alter believed that the community disapproves of adolescent childbirth), but lower if the alter personally believed that an older age for giving birth is desirable (PP 18% (95% CI = 17%-19%] compared to 24% (95% CI = 22%-25%) if the alter did not believe that an older age for giving birth is desirable). In our previous work [9] we found that village-level aggregated perceived norms towards the acceptability of adolescent childbirth and village-level proportion of women who had given birth as an adolescent were both strongly associated with a girl’s likelihood of having had an adolescent childbirth. In Model 2 we included these village level measures in the model and found that the association between alters’ perceived norms and adolescent childbirth was highly attenuated with the inclusion of the village level measures, but that the association between an alter’s adolescent childbirth and the girl’s adolescent childbirth outcome was not.

**Table 4:** Association of social network normative characteristics and having had an adolescent pregnancy among girls less than 21 y of age

	Model 1			Model 2		
	**Beta**	**SE**	***P*-value**	**Beta**	**SE**	***P*-value**
Village level AP norms				0.17	0.06	0.00
Village level AP female				0.16	0.06	0.01
Alter AP	0.54	0.04	0.00	0.48	0.04	0.00
Alter AP norms	0.14	0.05	0.01	0.06	0.05	0.21
Alter Attitude Pregnancy Age Older	-0.19	0.05	0.00	-0.17	0.05	0.00
Alter Attitude Pregnancy Age DK	-0.17	0.15	0.27	-0.14	0.15	0.35
Ego AP norms	0.34	0.14	0.02	0.26	0.15	0.07
Ego Attitude Pregnancy Age Older	-1.17	0.13	0.00	-1.16	0.13	0.00
Ego Attitude Pregnancy Age DK	-1.30	0.38	0.00	-1.23	0.38	0.00
Tie strength	-0.08	0.02	0.00	-0.07	0.02	0.00

SE – standard error, AP – adolescent pregnancy, DK – don’t know

**Table 5:** Association of social network normative characteristics and having had an adolescent pregnancy among girls less than 21 y of age*

	Alter AP			Alter norms				Alter attitude, older age				Alter attitude, don’t know		
	**Beta**	**SE**	***P*-value**	**Beta**	**SE**	**P**	**Beta**		**SE**	***P*-value**	**Beta**		**SE**	***P*-value**
Health advice give	**0.96**	**0.16**	**0.00**	0.17	0.20	0.38	0.12		0.20	0.54	-0.44		0.61	0.47
Trust borrow $	**0.38**	**0.09**	**0.00**	0.03	0.13	0.80	-0.11		0.13	0.41	-0.57		0.38	0.13
Personal private	**0.57**	**0.08**	**0.00**	**0.27**	**0.11**	**0.02**	**-0.34**		**0.11**	**0.00**	-0.24		0.35	0.50
Free time	**0.57**	**0.07**	**0.00**	**0.21**	**0.10**	**0.04**	**-0.26**		**0.10**	**0.01**	**-0.61**		**0.29**	**0.04**
Sibling	**0.78**	**0.12**	**0.00**	0.14	0.16	0.36	**-0.33**		**0.15**	**0.03**	-0.10		0.33	0.75
Closest friend	**0.70**	**0.08**	**0.00**	**0.23**	**0.11**	**0.04**	0.03		0.11	0.81	**-0.61**		**0.31**	**0.05**
Partner	**2.43**	**0.44**	**0.00**	0.30	0.24	0.22	-0.37		0.24	0.13	-0.46		0.63	0.47
Provider	**0.36**	**0.12**	**0.00**	**0.49**	**0.15**	**0.00**	**-0.66**		**0.15**	**0.00**	-0.01		0.46	0.99
Health advice get	**0.38**	**0.09**	**0.00**	0.19	0.12	0.09	**-0.18**		**0.10**	**0.09**	**-0.85**		**0.50**	**0.09**
Trust lend $	**0.70**	**0.11**	**0.00**	0.09	0.15	0.55	**-0.29**		**0.15**	**0.06**	-0.40		0.46	0.38
Town leaders	**0.26**	**0.10**	**0.01**	0.10	0.12	0.41	-0.08		0.11	0.49	-0.01		0.41	0.98
Father	0.08	0.22	0.70	0.29	0.26	0.26	**-0.45**		**0.23**	**0.05**	**-1.84**		**0.76**	**0.02**
Mother	**0.54**	**0.15**	**0.00**	0.27	0.18	0.13	**-0.42**		**0.16**	**0.01**	-0.33		0.67	0.63
Female alter	**0.58**	**0.05**	**0.00**	**0.15**	**0.07**	**0.04**	-0.08		0.07	0.25	-0.23		0.23	0.32
Male alter	**0.37**	**0.08**	**0.00**	**0.16**	**0.08**	**0.05**	**-0.28**		**0.08**	**0.00**	-0.22		0.23	0.34
Same household	**0.43**	**0.07**	**0.00**	**0.27**	**0.10**	**0.01**	**-0.37**		**0.10**	**0.00**	-0.41		0.29	0.17

SE – standard error, AP – adolescent pregnancy

*Stratified by type of relationship. All models include sociodemographic controls (not shown).

**Table 6:** Association of social network normative characteristics and having had an adolescent pregnancy among girls less than 21 y of age*

	Alter AP			Alter norms			Alter attitude, older age			Alter attitude, don’t lnow		
	**Beta**	**SE**	***P*-value**	**Beta**	**SE**	***P*-value**	**Beta**	**SE**	***P*-value**	**Beta**	**SE**	***P*-value**
**Health advice give**	**0.93**	**0.16**	**0.00**	0.10	0.20	0.61	0.13	0.20	0.53	-0.39	0.62	0.53
**Trust borrow $**	**0.34**	**0.09**	**0.00**	-0.04	0.13	0.78	-0.08	0.13	0.55	-0.53	0.38	0.16
**Personal private**	**0.52**	**0.08**	**0.00**	**0.19**	**0.11**	**0.09**	**-0.31**	**0.11**	**0.00**	-0.20	0.36	0.58
**Free time**	**0.51**	**0.07**	**0.00**	0.14	0.10	0.17	**-0.24**	**0.10**	**0.02**	**-0.59**	**0.30**	**0.05**
**Sibling**	**0.69**	**0.12**	**0.00**	0.04	0.15	0.81	**-0.30**	**0.15**	**0.04**	-0.09	0.34	0.79
**Closest friend**	**0.63**	**0.08**	**0.00**	0.13	0.11	0.22	0.06	0.11	0.60	**-0.56**	**0.31**	**0.07**
**Partner**	**2.43**	**0.44**	**0.00**	0.20	0.25	0.43	-0.36	0.24	0.14	-0.46	0.63	0.47
**Provider**	**0.34**	**0.12**	**0.01**	**0.37**	**0.16**	**0.02**	**-0.67**	**0.15**	**0.00**	0.01	0.45	0.99
**Health advice get**	**0.33**	**0.09**	**0.00**	0.12	0.12	0.29	-0.15	0.10	0.16	-0.78	0.51	0.13
**Trust lend $**	**0.64**	**0.12**	**0.00**	0.07	0.15	0.65	**-0.27**	**0.15**	**0.08**	-0.39	0.47	0.40
**Town leaders**	**0.18**	**0.10**	**0.07**	0.04	0.12	0.70	-0.03	0.11	0.80	0.01	0.42	0.98
Father	0.03	0.22	0.88	0.20	0.26	0.44	**-0.44**	**0.23**	**0.05**	**-1.74**	**0.77**	**0.02**
Mother	**0.48**	**0.15**	**0.00**	0.18	0.18	0.30	**-0.39**	**0.16**	**0.02**	-0.23	0.65	0.72
Female alter	**0.52**	**0.05**	**0.00**	0.07	0.07	0.34	-0.05	0.07	0.43	-0.19	0.23	0.42
Male alter	**0.32**	**0.08**	**0.00**	0.10	0.08	0.24	**-0.24**	**0.08**	**0.00**	-0.21	0.23	0.37
Same household	**0.40**	**0.07**	**0.00**	**0.18**	**0.10**	**0.07**	**-0.36**	**0.10**	**0.00**	-0.42	0.30	0.16

SE – standard error, AP – adolescent pregnancy

*Stratified by type of relationship and controlling for village level proportion of those who report that the community supports adolescent birth, and village level proportion of women who have had an adolescent birth. All models include sociodemographic controls (not shown).

We ran several interaction models (not shown), and found that the relationship between an alter’s adolescent childbirth and the likelihood of a girl’s adolescent childbirth was lower the older the alter was compared to the girl, lower if the alter was someone whom she herself nominated but who did not nominate her back, higher the greater the tie strength between her and the alter (the stronger the relationship), and, higher if the alter reported approving of a higher age of first childbirth. We also found that the association between an alter’s belief in an older age of first childbirth and a lower likelihood that the girl had had an adolescent childbirth was stronger if the alter was a male and if the alter was in the same household.

#### Girls’ own nominated network

**Figure 1:**
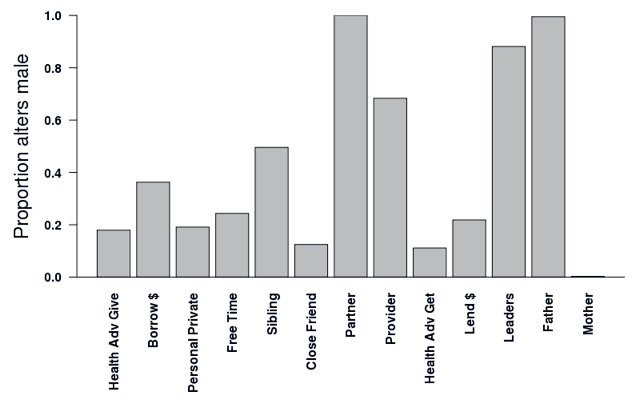
Proportion of alters nominated by adolescent girls 15-20 who are male across all name generators.

**Figure 2:**
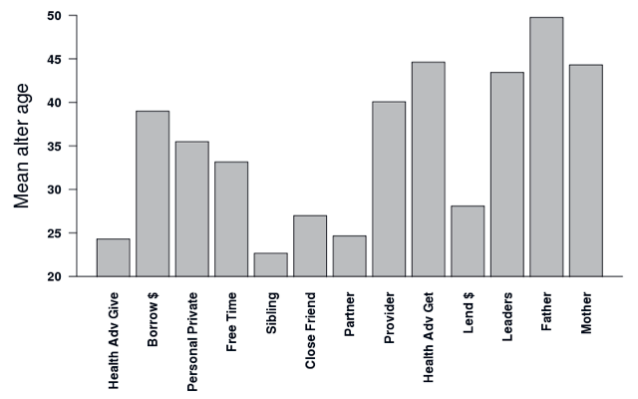
Mean age for alters nominated by adolescent girls 15-20 across all name generators.

In **Table 5**, we stratifed the models from Table 4 by the type of relationship, based upon only those the girl herself nominated and by which name generator was used to identify the relationship. As any one individual could be identified with multiple name generators, certain dyadic relationships will be present in the analyses for multiple name generator models. So, for instance, a mother/daughter dyad was represented in the mother model, but also possibly in the free time model or the personal private model. **Figure 1** and **Figure 2** illustrate the differences in alter characteristics depending upon the name generator. In **Figure 1** we see that alters tended to be female, with the exception of those who were male family members or town leaders. **Figure 2** illustrates the average age per alter. Not surprisingly, those to whom an adolescent girl goes for health advice (44 years old on average) or to borrow money (38 years old on average) tended to be older, while those to whom a girl gave advice (24 years old on average) or lent money (28 years old on average) were younger, although still older than she was.

**Figure 3:**
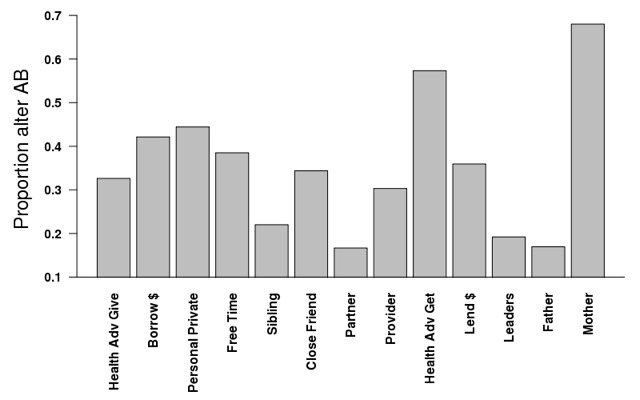
Proportion of alters nominated by girls 15-20 who have had an adolescent birth (AB) across all name generators.

**Figure 4:**
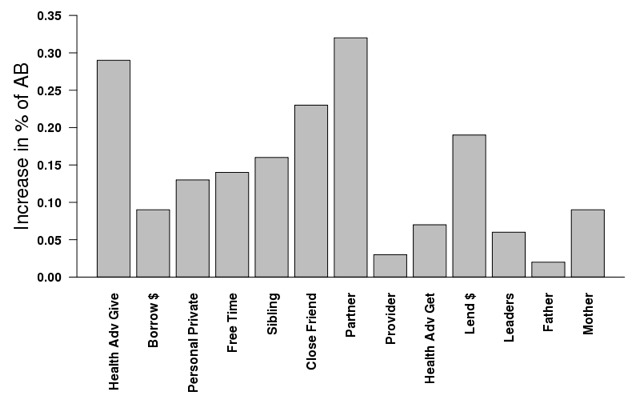
The increase in risk of adolescent childbirth among girls 15-20 conditional on whether their alter has had an adolescent childbirth (AB) across all name generators.

Each row represents the model from one name generator, controlling for all covariates (not shown). Across almost every type of relationship with the exception of *father*, if an alter had had an adolescent childbirth, the likelihood that the girl had had an adolescent childbirth was much higher compared to when the alter had not had an adolescent childbirth (column 1). **Figure 3** illustrates the proportion of alters who had had an adolescent childbirth across name generators. The proportion of mothers in this population of adolescent girls aged 15-20 years who had had an adolescent childbirth was notably high (68%), while the proportion of girls’ partners who had reported fathering a child as an adolescent was (15%) and the proportion of girls’ fathers who had fathered a child as an adolescent birth (15%) were both far lower. **Figure 4** shows the increase in probability that a girl had had an adolescent childbirth when the alter had had an adolescent childbirth compared to when the alters had not. Although the proportion of males in the sample who had had an adolescent childbirth was small, the likelihood of a girl having had an adolescent childbirth if her partner had was particularly high (increase of 34%), while there was no significant association between the adolescent childbirth of the girl and that of her father. On the other hand, although mothers had the highest rates of adolescent childbirth, the increase in likelihood that a girl had had an adolescent childbirth when her mother had was not particularly high (increase of 9%), while the increase in likelihood that a girl had had an adolescent childbirth when someone to whom she gives health advice had was notably high (30%).

In column 2 of **Table 5** we see that alters' perceived norms around community support for adolescent childbirth was significantly associated with girls’ adolescent childbirth when her alter was someone with whom she spent free time, discussed personal and private matters, or was a close friend or provider. We also found that when an alter reported a personal attitude supportive of a higher age of first childbirth, the likelihood that a girl had had an adolescent childbirth was lower; notable relationships were mothers, fathers, providers and siblings.

The bottom 3 rows of **Table 5** include analyses broken down by gender of alter, and same household status. We found strong relationships across all three measures for those in the same household, and for male alters. While our measure of proportion of the village who had given birth as an adolescent was restricted to women, given that they are far more likely to have had an adolescent childbirth, our results stratified by sex suggest that male adolescent childbirth was also an associated factor.

In **Table 6**, we reran the analyses from **Table 5** adding the village-level normative factors: proportion who think the community supports adolescent childbirth, and proportion of women who have had an adolescent childbirth. Column 1 results confirm what we found in our earlier models, which is that the relationship between an alter having had an adolescent childbirth and a girl’s adolescent childbirth outcome was not at all attenuated by including the village proportion of adolescent childbirth in the model. Both exerted an independent effect. However, across all types of alters, alters’ perceived norms of community support for adolescent childbirth were either not significant or significantly attenuated after including village-level adolescent childbirth in the models. In the case of provider, those with whom adolescent girls discussed matters personal and private, and those in the same household, the magnitude of effect diminished considerably, but was still significant (or close to significant in the case of same household, and personal private) (column 2).

## DISCUSSION

In this study, we considered the social network factors, both at the proximal interpersonal and at the distal community levels, associated with girls’ adolescent childbirth outcomes among the population of 176 villages in rural Honduras. Our primary aim was to understand the reference groups, or sources of normative support and pressure, for adolescent childbirth. Our findings show compelling support for the hypothesis that there are reference groups at multiple levels that may influence adolescent childbirth outcomes.

We found that having both a father in the village and a mother in the village had strong associations with a lower likelihood of giving birth as an adolescent. However, when we included the nomination of a spouse in the models, the role of the girl’s father remained, but her mother’s influence became insignificant. Our initial supposition was that girls who had had an adolescent childbirth may have left the village with a partner, and therefore the girl’s parents would not have been present because she most likely would have moved from her home village. To adjust for this, our models included a measure of years lived in the village, and we included a measure of spouse in the final model as well. Why are fathers still significant but mothers are not? In an exploratory analysis of factors associated with having named a spouse (not shown), girls who named a mother were three times less likely to have nominated a spouse than those who named fathers. Clearly the association with having a mother and an adolescent childbirth was accounted for statistically by partnership with a spouse but this was not the case for having a father. This is important evidence that fathers may exert a strongly protective effect against adolescent childbirth in this population, independent of whether or not the adolescent girl is partnered, whereas once the girl is partnered the influence of the mother in protecting against adolescent childbirth goes away. This is consistent with previous research showing that a missing father is an important risk factor for early childbearing [5].

We found evidence of strong normative dynamics at the interpersonal level that were associated with adolescent childbirth, with strong clustering in these networks. In terms of descriptive norms, the probability that a girl had had an adolescent childbirth if her alter did was high across all types of relationships with the exception of fathers, and was stronger when the relationship between the adolescent girl and the alter was stronger (higher tie strength). This is compelling evidence that interpersonal proximal network influences constitute a strong source of normative influence for adolescent childbirth. When we included the proportion of women in the village that had had an adolescent childbirth, a more distal source of potential influence, we found no notable reduction in the relationship between the girl’s adolescent childbirth and that of her alter. This suggests that the descriptive norm regarding the behaviour of others in the community to whom the adolescent girl is unrelated has little influence on her behaviour. The clustering of adolescent pregnancy was occurring at multiple levels: at that of her direct social contacts, and at the level of the greater community, with the association with proximal alters playing a much stronger role. However, with the question that asked individuals their perception of the community’s response to adolescent childbirth – exploring the influence of injunctive norms – we found a different dynamic. While we saw significant associations in the relationship between an alter’s perception of positive community norms towards adolescent childbirth and a girl’s own adolescent childbirth, most of those relationships diminished or were no longer significant after we included the village-level aggregate norm in the model. This suggests that injunctive norms are most salient at the level of the community and less salient at the level of direct social contacts, and that the reference group for sources of injunctive normative pressure may be mostly occurring within the village context, more so than within individual relationships. The fact that community aggregate norms were more strongly associated than individual alter norms provides us evidence for a causal relationship: it is unlikely an entire community would change its perception of norms around childbirth in response to any one girl’s childbirth, while for individual alters this could be a reasonable consideration.

We did not include the aggregated attitudes towards best age for childbirth in the models because our previous research found it to be non-significant [9]. However, individual attitudes were strongly associated, particularly for familial relationships, those in the same household, and those who the girls considered close friends or whom they trusted to discuss personal and private matters. Interestingly, girls were more likely to have had an adolescent childbirth when they nominated an alter who had had an adolescent childbirth, but who reported approving of a higher age of first birth. In this case the disconnect between what the alter reported as their own attitude vs their own previous experience was associated with a strong likelihood of adolescent childbirth among this population of girls. Because we have cross-sectional data we cannot track these dynamics through time. Thus, it is possible that the alter could, having had an adolescent childbirth themselves, and having observed the adolescent childbirth experience of the girl, decided that an older age of childbirth is optimal and reported accordingly. We cannot say then whether these attitudes work for or against adolescent childbirth. It is also possible that the discordance between alters’ stated attitude (preference for older age at first childbirth) and their behaviour (adolescent childbearing) reflects a “taboo gap” wherein the topic of sex and early childbearing is not something that was discussed openly, even among adolescent girls in our study population with their alters. This may be similar to the situation we observed in Zambia, where adolescent girls in communities with high levels of discordance – in this case among adults – between attitudes toward premarital sex and the behavior of premarital sex was associated with significantly increased risk of HIV acquisition (Weber et al, 2019). In both cases, the lack of care-seeking for contraception in response to the taboo gap may have contributed to pregnancy in Honduras and increased HIV prevalence in Zambia among adolescent girls.

Research on social network influences invariably generates hypotheses, but ultimately we aim to uncover cause and effect relationships. We would like to know the probable causes both for general scientific purposes and to give us strategic points for intervention. With cross-sectional data we cannot make causal claims. However, with strong cross-sectional data we can uncover crucial clues for causality, and areas for which expensive longitudinal data would be worth the investment. In this analysis, we have interesting evidence for attitudes at the alter level associated with adolescent childbirth, but little to help us decipher the directionality of those associations. We have reasonable evidence that village-level community norms around adolescent childbirth may exert influence. However, we have very compelling evidence that adolescent childbirth both in a girl’s proximal networks, and in her wider community, normalises adolescent childbirth, allowing it to persist through generations. While a girl, having had an adolescent childbirth, may choose new relationships with other girls and women who have had adolescent childbirths, this is unlikely to be the case across her entire network, and certainly not possible in the case of direct family members. Furthermore, it is extremely unlikely that girls with low levels of education and minimal financial resources are choosing villages with high overall rates of adolescent childbirth after they themselves have had one. Of all the social factors we have considered, exposure to adolescent childbirth within her network seems to be the most salient possible determinant, regardless of the norms or attitudes that oppose it. In contrast, the presence of a father in her life emerges as a strong deterring factor.

This analysis has several limitations. The first is that the data are not longitudinal so we cannot track the influence of norms and attitudes across time. Second, this population of girls (aged 15-20) included those who may still have an adolescent birth, and yet statistically they fell into the category of those who have not. Moreover, adolescent girls in both groups – those who have had and those who have not had an adolescent childbirth – may have had additional pregnancies which ended in miscarriage, abortion or stillbirth which are not included in our analysis. Third, while rates and demographic determinants of adolescent childbirth in this population were consistent with the determinants in Latin America in general, this population is specific to a certain region in Honduras, and so some factors may not be generalisable. Finally, while these results may offer important insights for contexts with low parental social control of adolescent sexual activity, they are not likely to be relevant in contexts where parental control is strong, and adolescent childbearing is often the result of arranged marriages.

## CONCLUSION

Adolescent childbirth is a serious issue in Latin America, affecting nearly one-third of the adolescent girls in our sample, and is becoming a high public health priority, both in Latin American and globally [2,44]. The question at hand, of course, is how to address it in an effective manner. In this study we considered factors associated with adolescent childbirth in a context of low parental supervision of adolescent sexuality. Girls were not having children because they were forced into marriage by their parents, but because they themselves chose partners with whom they were sexually active at a young age. We already know that adolescent childbirth in low parental control contexts like Latin America is associated with a consistent cluster of challenging factors, including lower levels of education, lower levels of income, and family instability, including absence of the father [2]. Our study indicates that while injunctive norms may play a role, they are not operating at a strong enough level to counter the descriptive norms: adolescent childbirth in the wider community and particularly in the proximal social network seems to be the prevailing force of influence. The challenge is to encourage norms that prevent adolescent childbirth without stigmatising those who have already gone through it. The shift in norms may have to happen both at the interpersonal level and at the community level, as both are potentially salient reference groups for adolescent childbearing norms. Programmatic efforts to counter the prevailing norms that limit a woman’s role to motherhood, and that support and encourage strong norms for girls’ education may play an important role in addressing this problem. Furthermore, greater understanding of the roles of fathers, and encouraging norms of positive and active fatherhood could be a powerful measure to reduce the family instability that is associated with adolescent childbirth. Our research suggests avenues for intervention, both in Latin America as well as other contexts in which low parental control of adolescent sexuality facilitates adolescent childbearing. However, the issue is undoubtedly complex, so intervention efforts will need to carefully consider and monitor the many contributing factors.

## Additional material

Online Supplementary Document
