# Supplementary material for: Social normative and social network factors associated with adolescent pregnancy: a cross-sectional study of 176 villages in rural Honduras
Source: J Glob Health. 2020 Mar 30;10(1):010706. doi: 10.7189/jogh.10.010706 (PMC7182389; doi:10.7189/jogh.10.010706)
Supplement: Online Supplementary Document [file jogh-10-010706-s001.pdf]

Demographic measures

We measured respondent age in years. Marital status was assessed by asking whether or not the respondent was married or in a civil union. Respondent religion was measured as “Catholic”, “Protestant”, “No religion” or “Other”. We included a validated discrete measure of respondent income sufficiency which asked respondents to rate how well their income met the needs of their families (Teaching, 2014): “It is not sufficient and there are major difficulties" was coded as 1,"It is not sufficient and there are difficulties was coded as 2,"It is sufficient, without major difficulties" was coded as 3, and "There is enough to live on and save was coded as 4. Education was coded on a scale of 0-9: "Have not completed any type of school" coded as 0,"1st grade" coded as 1,"2nd grade" coded as 2,"3rd grade" coded as 3,"4th grade" coded as 4,"5th grade" coded as 5,"6th grade" coded as 6,"Some secondary" coded as 7,"Secondary" coded as 8,"More than secondary" coded as 9. We included a measure of self-identified indigenous status (yes or no). Food security was the sum of 4 yes/no questions: (1) In the past 3 months, for lack of money or other resources, did you ever worry that your household would run out of food? (2) In the past 3 months, for lack of money or other resources, did your household ever run out of food? (3) In the past 3 months, did you or any adult in your household eat only one meal in a day or go without eating for a whole day because of lack of money or other resources to get food? and, (4) In the past 3 months, did any child in your household eat only one meal in a day or go without eating for a whole day because of lack of money or other resources to get food? Respondents were also asked the duration of their time in the village with answer choices “Since birth”, “More than 1 year”, or “Less than 1 year” used categorically. We asked each respondent to provide the number of years they had been living in the village.

NAME GENERATOR QUESTIONS

Does your mother live in this town?

1.) **If yes, what is the name of your mother?**

Does your father live in this town?

2.) **If yes, what is the name of your father?**

What are the names of your siblings over the age of 12 that live or work here?

Do you have any children who don't live with you but do live in this village over the age of 12?

3.) **What are their names?**

Are you married or living in a civil union?

4.) **What is the name of your partner?**

In the next section, we will ask you some questions about who are the people that you do different things with, and to name those people specifically. You can answer the questions with names of people who are OVER THE AGE OF 12 and WHO CURRENTLY LIVE OR WORK IN THIS VILLAGE ONLY. These people may include any friends, family, people you work with, people that work for you, neighbors, etc. When you answer these questions, you may answer with one person, more than one person, or say there is no one. You are free to include up to 5 people.

5.) **Who do you trust to talk to about something personal or private? (including friends, family, people you work with, neighbors etc, who live or work in this village)**

6.) **With whom do you spend free time? (including friends, family, people you work with, neighbors etc, who live or work in this village)**

7.) **From whom would you feel comfortable asking to borrow 200 Lempiras if you needed them for the day? (including friends, family, people you work with, neighbors etc, who live or work in this village)**

8.) **Who do you think would be comfortable asking you to borrow 200 Lempiras for the day? (including friends, family, people you work with, neighbors etc, who live or work in this village)**

9.) **Who would you ask for advice about health related matters? (including friends, family, people you work with, neighbors etc, who live or work in this village)**

10.) **Who comes to you for health advice? (including friends, family, people you work with, neighbors etc, who live or work in this village)**

11.) **Besides your partner, parents or siblings, who do you consider to be your closest friends? (including friends, family, people you work with, neighbors etc, who live or work in this village)**

12.) What are the names of the towns leaders?

We have asked questions about the people who are positive connections in your life. Our study aims to group people that get along well. Now we are going to ask you one question about the people in your life with whom you usually do not get along well. It is important that you understand that we will keep all of your responses secret and that they will only be used for the purpose of mathematical analysis. Your information will never be shared with anyone.

13.) Who are the people in this town with whom you do not get along well? (including friends, family, people you work with, neighbors etc, who live or work in this village)

Do you have a patron/patrona?

14.)**What is his/her name?**

Data collection

Geographic Maps

The first stage in data collection was making geographical maps of each village. The maps serve as a means of quality control and help focus the efforts of each survey team. The mapmakers used satellite imagery to get an idea of the town's layout, after which the team went into the village and placed every edifice on the layout. Once the map was completed every structure was given a unique code that enumerators used when conducting census and surveys

Photographic Census

After the map of a town was complete, enumerators visited each building and ask to photograph each person living in the residence. These pictures were taken on tablets preloaded with *Trellis*, a tablet-based survey program designed to facilitate network data collection in the field. The pictures and some preliminary demographic information were saved to the program's database so the pictures could used in the next stage to increase the accuracy and efficiency of network data collection. We used photo identification primarily to solve issues of unique identification in the presence of name similarity and high illiteracy. During previous data collections efforts in this area, the research team learned that in one town fifteen women were named Maria Hernandez. And so, when a respondent answers that Maria Hernandez is one of her friends, enumerators would have been stymied without additional information to specifically identify one Maria from the others. Past efforts have measured the additional information by asking qualifying questions about Maria; however this increases the time it takes to gather data while decreasing accuracy. Pictures are faster and more accurate.

Large Survey and Network Data Collection

Upon completing the photographic census, we undertook the baseline survey in each village. This survey included the battery of "name generators" that served to identify the social alters, thereby building each villages' social network[10]. Name generators covered a broad grouping of relationship types, including affective, kinship, and resource exchange. To uniquely identify the connections between individuals, we used the photographs collected in the census.
